# The Effect of Land Use Change on Transformation of Relief and Modification of Soils in Undulating Loess Area of East Poland

**DOI:** 10.1155/2014/341804

**Published:** 2014-12-31

**Authors:** Jerzy Rejman, Anna Rafalska-Przysucha, Jan Rodzik

**Affiliations:** ^1^Institute of Agrophysics, Polish Academy of Sciences, Str. Doświadczalna 4, 20-290 Lublin, Poland; ^2^Faculty of Earth Sciences and Spatial Management, University of Maria Curie-Skłodowska, al. Kraśnicka 2c, 20-718 Lublin, Poland

## Abstract

The change of primary forest areas into arable land involves the transformation of relief and modification of soils. In this study, we hypothesized that relatively flat loess area was largely transformed after the change of land use due to erosion. The modifications in soil pedons and distribution of soil properties were studied after 185 years of arable land use. Structure of pedons and solum depth were measured in 128 and soil texture and soil organic carbon in 39 points. Results showed that soils of noneroded and eroded profiles occupied 14 and 50%, respectively, and depositional soils 36% of the area. As a consequence, the clay, silt, and SOC concentration varied greatly in the plowed layer and subsoil. The reconstructed profiles of eroded soils and depositional soils without the accumulation were used to develop the map of past relief. The average inclination of slopes decreased from 4.3 to 2.2°, and slopes >5° vanished in the present topography. Total erosion was 23.8 Mg ha^−1^ year^−1^. From that amount, 88% was deposited within the study area, and 12% was removed outside. The study confirmed the hypothesis of the significant effect of the land use change on relief and soils in loess areas.

## 1. Introduction

Any change in land use or even in crop rotation affects the intensity of water or tillage erosion, the processes that provide redistribution of soil material and changes in soil properties. However, the scale of soil changes in space and time is weakly recognized. Loess areas with high soil erodibility, long lasting arable land use, and rolling landscape belong to the regions of the highest water erosion risk in Europe [1]. Mechanical tillage has increased the human pressure on soil and started to constitute an important part of total erosion. While water erosion dominated on longer slopes, soil translocation due to tillage transformed the upper areas of cultivated hillslopes [2–4].

Erosion and deposition resulted in a large modification of soils developed from loess. To describe the changes, Turski et al. [5] proposed a classification of soils based on reduction of pedon of Haplic Luvisols. The classification included noneroded soils, four classes of erosion, and depositional soils. To distinguish erosion classes, the illuvial horizon B was separated in three parts (Bt1, Bt2, and BC) that were easily recognized in the field by the identification of the B subhorizon located below the plowed layer. The studies showed a mosaic type of localization of noneroded, eroded, and depositional soils located on slopes and within the plateau of the Lublin Upland [5–7]. Houben [8] showed a similar lack of spatial pattern in location of soils in loess catchment of Southern Germany. One of the reasons for uncertainty in localization of soils could be a large density of open and closed depression in loess areas and different degree in filling of the depressions by deposited material. The density of depressions is assessed within the range from 1 to 16 per km^−2^ [9, 10]. The modification of pedons resulted in redistribution of soil material and changes in soil properties [6, 11–13].

Analysis of spatial distribution of soil properties is important for evaluation of landscape evolution after the change of land use from natural forest into arable land [8, 14]. The studies concern the comparison of present and past topography and try to find a rate of landscape transformation. Topography of the past is reconstructed on the basis of point-measurements of erosion and deposition, and the studies enable calculating erosion budget for catchments [15, 16]. Radionuclide caesium 137 (^137^Cs) is the most widespread method in evaluation of distribution of erosion and deposition. Description of soil profiles is weakly represented and needs an accurate description of noneroded soil profiles and establishment of the relations between soil depth and topographical features, that is, slope, aspect, upslope area, relative height difference, and so forth [17–19]. However, the studies did not give a clear response of the relations [8, 20, 21]. The assumption that erosion processes under natural forests did not introduce significant changes to soil profiles was the basis of reconstruction of the past relief. Studies under old forest in loess areas showed that soil profiles were not affected by erosion outside the gullied areas and confirmed the assumption [20, 22].

Previous studies in loess belt of the Lublin Upland were limited to recognition of the state of soil profile modification and its effect on soil properties [5–7]. However, evaluation of the changes that were introduced to soil and relief needs more comprehensive attitude that should also include a reconstruction of the past topography and analysis of changes in relief.

The purpose of the studies was evaluation of the changes that were introduced to the relief and soils after 185 years of arable land use of loess area. In this study, we hypothesized that relatively flat loess area at present was largely transformed due to erosion and deposition. The hypothesis was tested by assessment of changes in the depth of soil solum and soil properties, and geostatistical techniques were used to study the spatial pattern of soil attributes. Description of soil profiles in relation to the soil position in the landscape was used to reconstruct the depth of eroded profiles and the past topography. The reconstruction of the past topography was used to calculate erosion and deposition and evaluation of slope transformation in the studied area.

## 2. Methods

### 2.1. Site Description

The study area is located in the northern part of the Lublin Upland (51°19′53′′N latitude, 22°23′19′′E longitude). Loess deposits in this part of the region are about 10–30 m thick. The mean annual rainfall is 550.6 mm and the mean daily air temperature 7.4°C. Both precipitation and temperature are not uniformly distributed over the year. The highest monthly precipitation of 81.7 mm occurs in July and the lowest of 23.4 mm in January. Snow represents about 15% of total precipitation, and the average duration of snow cover is about 90 days. Annual rainfall erosivity evaluated on the basis of the *R*-factor of the USLE [23] is relatively low and ranges from 590 to 1850 MJ mm ha^−1^ h^−1^ [24]. The mean air temperature ranges from −3.4 in January to 17.9°C in July. Dry forest (*Tilio-Carpinetum*) was a natural vegetation of the area [25], and Haplic Luvisols developed from loess were typical soils [26].

**Figure 1:**
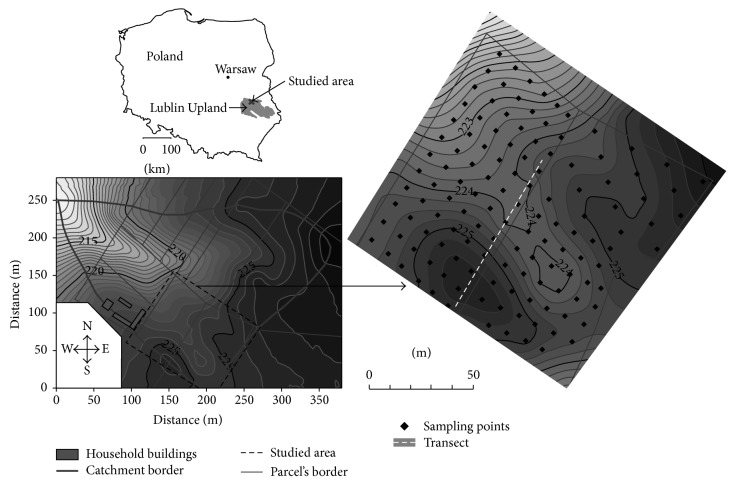
Location of the study area and localization of sampling points for soil profiles analyses and soil transect.

The study area of 1.3 ha occupies part of two parcels and is located near the watershed zone in the southern part of small catchment of dry basin type (Figure 1). The study area represents a rolling type of relief with relatively small differences of relative height that ranges from 221 to 226 m a.s.l. The site was deforested in 1820 and from that time remains under arable land use. The change of land use was reflected on topographical maps from 1804 and 1830. Present arrangement of parcels was established after land consolidation in 1932. Typical crop rotation included sugar beets (*Beta vulgaris* L.) or potatoes (*Solanum tuberosum* L.), spring barley (*Hordeum vulgare* L.) or spring wheat (*Triticum aestivum *L.), red clover (*Trifolium pretense* L.), and winter wheat (*Triticum aestivum* L.) or, earlier, rye (*Secale cereale* L.). In the early 1990s, red clover was removed from the rotation. Tillage included a mouldboard ploughing once or twice a year, and a chisel ploughing in spring. Mouldboard ploughing is performed to the depth of 25 cm in the autumn, and to 15 cm after cereal harvest, the same depth as chisel. Mechanical tillage has been started from the 80s of the last century.

A detailed topographic survey of the study area was carried out with a tachymeter in the autumn of 2005. On the basis of measurements, topographic maps were produced with Surfer version 10 [27]. Analysis of map showed that inclination of slopes varied from 0 to 5° with a mean of 2.2° within the study area.

### 2.2. Soil Characteristics

Thickness of soil horizons was established on the basis of description of intact soil cores of 2.5 cm diameter. Soil cores were taken to the depth of calcareous loess in noneroded and eroded soils, and to the lower border of BC horizon in depositional soils. The horizons were separated by difference in soil color and consistency. On the basis of measurements, depth of soil solum, that is, the cumulative thickness of soil horizons from Ap to BC, was established. The border between decalcified and calcareous loess was determined with 10% HCl. The soils were classified according to Turski et al. [5]. Noneroded soils characterized the full sequence of horizons typical for Haplic Luvisol, that is, Ap-E-Bt1-Bt2-BC-C-Ck. With reduction of soil profile, three erosion classes were distinguished. Slightly eroded soil characterized the loss of E horizon, and the profile had the following sequence: Ap-Bt1-Bt2-BC-C-Ck. With the loss of deeper horizons, the soils were classified as moderately (Ap-Bt2-BC-C-Ck) and severely (Ap-BC-C-Ck) eroded. Separation of the illuvial B horizon in three parts (Bt1, Bt2, and BC) is a difference to the standard description of Haplic Luvisol provided by WRB [26] and the Polish Soil Systematic System [28]. In this study, in contrast to previous work of Turski et al. [5], all soils with accumulated soil material that overlay the original soil were classified as depositional soils. Generally, the sequence of horizons of depositional soils was Ap-C1-Ab-E-Bt1-Bt2-BC-C-Ck. Contribution of soils in the study area was calculated as a percentage of soils to the total number of soil cores.

Samples for analysis of soil texture and soil organic carbon (SOC) were taken from the plowed layer (0–26 cm) and subsoil (26–50 cm). The samples were air-dried and sieved on 2 mm mesh. The particle size distribution was determined by areometric method [29], and SOC by wet combustion with dichromate solution [30].

Sampling for profile description and soil properties was performed in a regular grid of 20 m × 20 m (in total 39 measuring points). Then, density of sampling for profile description was increased and adjusted to the expected changes in soil redistribution that were visible by change of color of the plowed layer. Finally, sampling was performed in a grid 10 m × 10 m for majority of the studied area, and the total number of soil cores was 128. Location of sampling points was established with a type on the basis of the measured distance from the borders of parcels. Sampling was performed in the autumn of 2003 and 2004. Additionally, distribution of soil depth was studied along a transect located along the border of two parcels (Figure 1). Within the transect of the total length of 85 m, 4 ditches of the length from 9 to 18 m were made to the depth of 2 m. The ditches were separated by a distance of about 10 m.

### 2.3. Reconstruction of the Past Relief and Calculation of Erosion and Accumulation

Elaboration of the map without accumulated layer in depositional soils was the first step in reconstruction of the past topography. To develop a map, the depth of deposition was subtracted from the relative height in each of the sampling points. The map was developed with Surfer using kriging with linear model. The soils were separated according to the position in the landscape in four groups: soils in top of hill's position, flat area, on slopes of the southern and northern exposition, and the floor of depressions. Then, the depth of solum of noneroded and buried soils was analyzed for separated locations. Mean depth of solum of the soils was used to reconstruct the depth of soils with eroded profiles. In the reconstruction, the depth of the past plowed layer Ab was assumed on 13 cm. To minimize the effect of possible errors, the reconstruction of profiles was limited only to the depth of the first disturbed horizon. It means that only depth of E and Bt1 was reconstructed for slightly eroded soils, and depth of deeper horizons was accepted without any changes. Similar procedure was used for moderately and severely eroded soils; that is, for the former the depth of E, Bt1, and Bt2 and for the latter the depth of E, Bt1, Bt2, and BC were reconstructed. The calculations were made in Excel. Depth of lost part of reconstructed profiles was added to the relative height in measured points. Then, a map of the past topography was developed on the basis of relative heights of reconstructed profiles and profiles without deposition. The map was produced using kriging with linear model, and distribution of slopes was analyzed. Amount of erosion was calculated from the difference in the depth of reconstructed and preserved soil profiles and deposition from the depth of accumulated material within the studied area.

### 2.4. Statistical Analyses

Experimental data were statistically analyzed using Statistica version 8 [31]. Geostatistical analyses were performed with GS+ [32, 33]. Selection of variograms was based on residual sums of squares (RSS) and determination coefficients (*R* ^2^). Semivariance models were used to produce soil maps with Surfer version 10 [27]. Before the performance of geostatistical analyses, the data were checked for trend and normality. A uniform lag interval of 10 and 20 m was used in geostatistical analyses for soil properties and solum depth, respectively. Rotation of semivariance was tested independently.

## 3. Results

### 3.1. Soil Erosion Classes and Depth of Soil Solum

Measurements of intact soil cores showed that the processes of soil erosion and deposition largely modified the soils within the studied area. Depositional and slightly eroded soils were the most frequent and their contribution amounted to 36 and 30% of the total number of cores, respectively. Noneroded soils were represented by 14, moderately eroded soils by 12, and severely eroded soils by 9% of cores.

**Figure 2:**
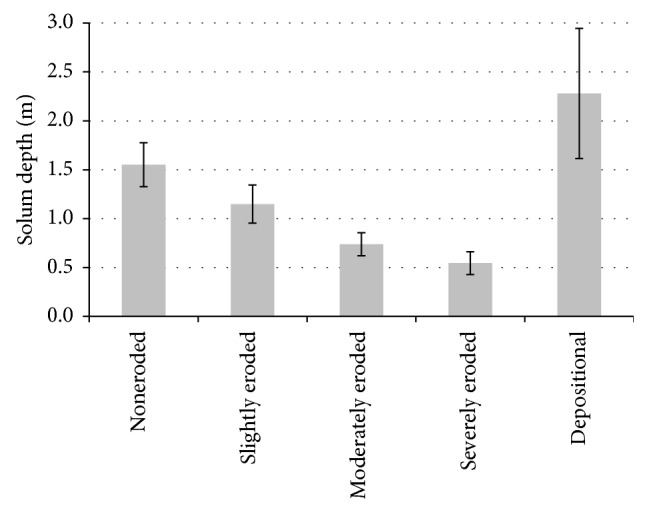
Mean depth and standard deviation of soil solum (Ap-BC) of the studied soils.

Taking into account all the studied soils, depth of solum (Ap-BC) ranged from 0.50 to 4.61 m with a mean of 1.52 and coefficient of variation (CV) of 50.9%. Depth of solum of noneroded soils ranged from 1.28 to 1.85 m, with a mean of 1.55 cm. Erosion and loss of parts or the whole soil genetic horizons resulted in decrease of mean depth of solum to 1.15, 0.74, and 0.55 m in slightly, moderately, and severely eroded soils, respectively (Figure 2). The differences in soil depth among the studied soils were statistically significant.

All the studied soils have similar depth of the plowed layer (Table 1). Assuming the depth of horizons of noneroded soils as a standard, the mean profile of slightly eroded soil was reduced not only by the loss of the whole E horizon, but also by the loss of 48% of Bt1 horizon. Moderately eroded soils characterize the loss of the E, Bt1 and 38% of Bt2 horizon, and severely eroded soils the loss of E, Bt1, Bt2 and 7% of BC horizons. Depth of decalcified layer C was similar among the studied soils and ranged from 0.04 to 0.23 m with a mean of 0.10 cm. Decalcified layer of soil overlaid the calcareous loess.

Depositional soils characterized the largest differentiation of solum depth that ranged from 1.2 to 4.61 m with a mean of 2.28 m. Three groups could be distinguished within depositional soils: initial, typical, and disturbed (Table 2). The initial group of depositional soils included the soils of deeper plowed layer than the depth of plow at present. Present and older plowed layer differed by soil consistency; however they had similar dark grey color. Soils of this group represent a transient form between noneroded and typical depositional soils. Larger depth of plowed layer indicates that accumulation of soil material has started in the locations of these soils. Separation of this group enables a more precise determination of the zones of accumulation. Depth of solum of the soils ranged from 1.2 to 2.16 m, and after substraction of the depth of present plowed layer Ap, it ranged from 0.95 to 1.86 m. Typical depositional soils consisted of soils where accumulated material covered the buried soils of full sequence of horizons characteristic of Haplic Luvisols. Present and buried plowed layers were separated by accumulated material that differed by color. Depth of solum of these soils ranged from 1.62 to 4.61 m, and thickness of accumulated material (Ap-C1) from 0.1 to 1.51 m with a mean of 0.53 m. Depth of old plowed layer Ab ranged from 0.07 to 0.34 with a mean of 0.14 m and coefficient of variation (CV) of 46%. Disturbed depositional soils consisted of profiles where accumulated material was settled on previously eroded soils. Three profiles represented this group, one with the loss of Ab and two others with the loss of Ab, E, and part of Bt1 horizon. Depth of solum of the disturbed depositional soils ranged from 1.62 to 2.06 m.

**Figure 3:**
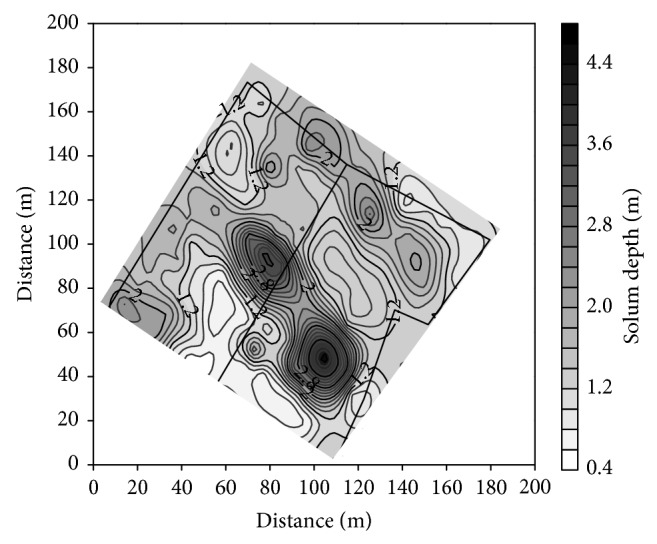
Distribution of soil solum (Ap-BC).

Distribution of soil solum showed a spatial structure of variability within the study area. The structure was best described by spherical isotropic model of semivariance with a range of autocorrelation of 31.9 m, nugget and sill of 0.0001 and 0.28 m^2^, respectively. The solum data were fitted to the model with coefficient of determination *R* ^2^ = 0.87, and anisotropy ratio was 1.75. Distribution of solum depth was presented in Figure 3.

### 3.2. Soil Texture Fractions and Soil Organic Carbon

The modification of pedon of Haplic Luvisols resulted in differentiation of soil texture and SOC concentration in both the plowed layer and subsoil of the studied soils. The most significant changes concerned the clay, silt, and SOC (Table 3). The clay concentration was the smallest in the plowed layer of noneroded soil and gradually increased in slightly, moderately, and severely eroded soils. The latter contained 2 times more clay than the plowed layer of noneroded soil. In subsoil, the largest concentration of clay was found in slightly eroded soil, and then it gradually decreased in moderately and severely eroded soil. Generally, the clay content in subsoil was larger than in the plowed layer of the studied soils. Severely eroded soil was the exception, as it contained more clay in the plowed layer in comparison to the subsoil. Silt concentration showed a reverse relation to the soil erosion class in comparison to the clay. The largest concentration of SOC was in the plowed layer of depositional and noneroded soils, and the smallest in severely eroded soil. The SOC concentration in subsoil of depositional soils was 2-fold larger in comparison to the others.

Clay and SOC concentration characterized the largest variation in the plowed layer, and the variation of both parameters in subsoil was even larger (Table 4). Content of clay was negatively linearly correlated with silt, and coefficients of determination *R* ^2^ were 0.91 and 0.97 in the plowed layer and subsoil, respectively. All studied soil properties showed a spatial structure of variability. Isotropic spherical models of semivariance with anisotropy ratio below 2.3 best described the structure (Table 4). The data were better fitted to the models for the plowed layer than subsoil, and determination coefficients *R* ^2^ ranged from 0.45 to 0.95. Generally, the ranges of autocorrelation for textural fractions varied from 32 to 36 m in the plowed layer and from 27 to 32 m in subsoil (apart of sand). The ranges of autocorrelation for SOC in the plowed layer and subsoil were 42 and 32 m, respectively. The calculated semivariograms were used in point ordinary kriging to produce the maps of clay and SOC distribution.

**Figure 4:**
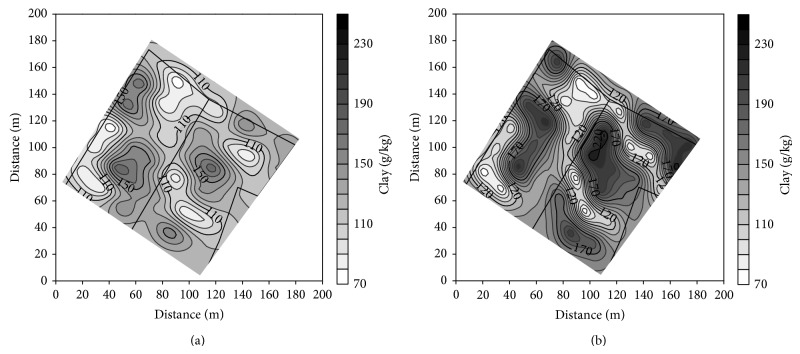
Distribution of clay (g kg^−1^) in the plowed layer (a) and subsoil (b).

**Figure 5:**
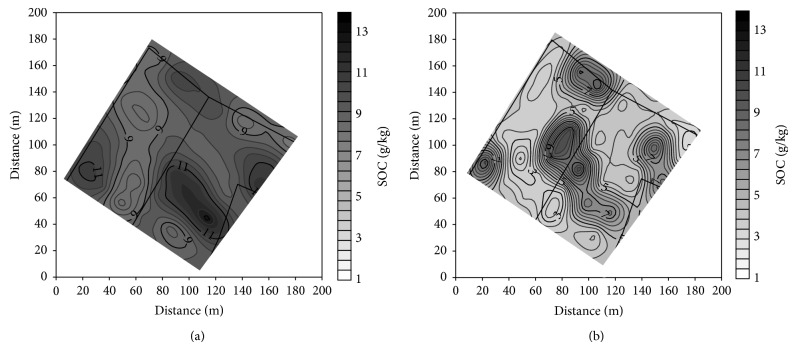
Distribution of SOC (g/kg) in the plowed layer (a) and subsoil (b).

Distribution of clay in the plowed layer well corresponds to the distribution of soil solum. The zones of high content of clay are the zones of shallow soils (Figures 4(a) and 2). The similarity is disturbed in subsoil, where only part of the zones of high clay content covers the area of shallow soils (Figures 4(b) and 2). The zones of the smallest content of clay correspond mainly with the area of deep depositional soils. Distribution of silt showed a reverse relation to the soil depth (not showed in the paper). Distribution of SOC is also related with distribution of soil solum (Figures 5 and 2). The relation between the zones of high SOC concentration and deep soils is better visible in subsoil than in the plowed layer.

### 3.3. Reconstruction of the Past Relief and Balance of Erosion and Deposition

**Figure 6:**
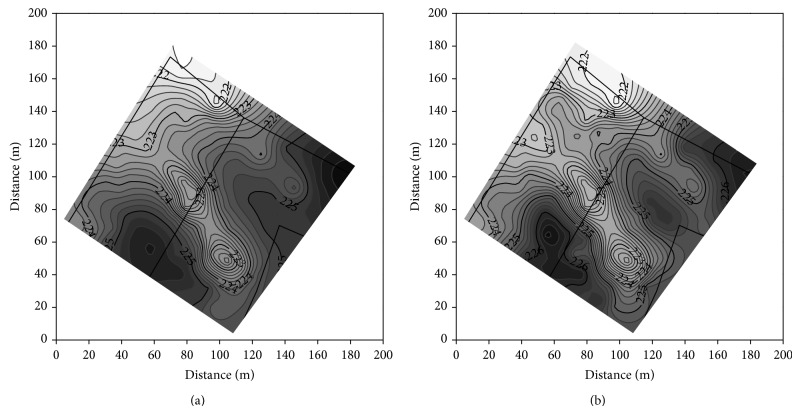
Topography of studied area without accumulated soil material (a) and the past relief (b).

Topography of the studied area without accumulated material was presented in Figure 6(a). Noneroded and buried soils (i.e., depositional soils after removal of deposition layer) of the full sequence of horizons typical for Haplic Luvisol were represented by 60 profiles. From that amount, soils located at hill top position were represented by 2, at flat area by 10, at slopes of N exposition by 14, at slopes of S exposition by 18, and at floor of depressions by 16 profiles (Table 5). Statistical analysis showed that the soils could be separated in 3 groups. The first group consisted of soils located at hill top position and slopes of southern aspect. The depth of the former ranged from 0.95 to 1.13 m, and the latter from 1.09 to 1.54 m. The second group consisted of soils located on relatively flat areas and slopes of northern aspect. The depth of solum of these soils ranged from 1.28 to 1.74 and from 1.51 to 1.86 m, respectively. Finally, the third group represented the deepest soils located at the floor of open and closed depressions, and the solum depth varied from 1.68 to 2.68 m. Soils with eroded profiles were classified at the same way. Eroded soils at flat position were represented by 17 profiles, and soils on slopes of southern and northern exposition by 20 and 26 profiles, respectively. Lost depth of eroded soils was calculated from the difference of the mean depth of noneroded and buried soils and preserved depth of eroded soils. The calculations were made taking into account the position of the soils in the landscape. The difference was added to the present relative height of the eroded sites. Then, the map of the past topography of the site was developed. The map is based on the reconstructed profiles and profiles without soil accumulation (Figure 6(b)).

Database of reconstructed and overbuilt profiles enabled calculating the amount of erosion and accumulation within the study area. Sum of accumulated and eroded soil material for all studied profiles was 29.52 and 32.59 m, respectively. The amounts divided by total number of profiles showed that the average profile was overbuilt by 0.23 m and eroded by 0.26 m. Assuming that soil density is 1.3 Mg m^−3^ [13] total erosion was 4394 Mg. From that amount, 88% was deposited within the study area, and 12% removed outside. Taking into account the period of arable land use of the area (185 years), total soil erosion was 23.8 Mg ha^−1^ year^−1^ in the studied area, that is, 1.41 mm year^−1^.

### 3.4. Transformation of Slopes of the Past Relief

**Figure 7:**
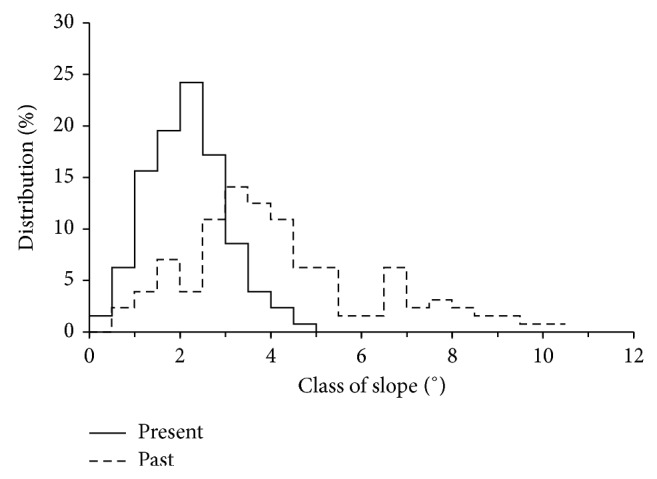
Distribution of slope classes in the present and past relief.

Analysis of slopes reflects the changes between present and past relief (Figure 7). Slopes in the range from 2.5 to 4.5° prevailed in the past topography, whereas slopes from 1 to 3° at present. In the latter, slopes above 5° were absent, whereas these slopes amounted to 30% of total slopes in the past relief. The average inclination of slopes decreased by about a half, that is, from 4.3 to 2.2° at the past and present topography.

### 3.5. Location of Soils in Transect

**Figure 8:**
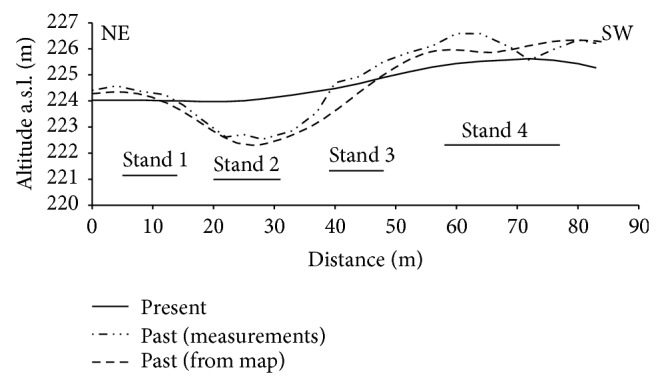
Present and past topography of the transect in the study area with localization of four soil pitches.

**Figure 9:**
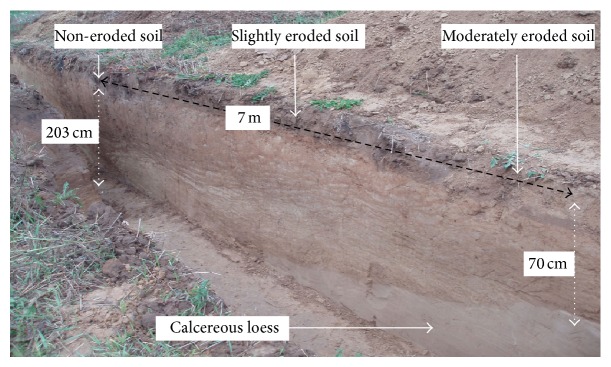
Changes in the depth of soil in transect at the distance of 66–70 m (part of the stand 4) (Photo J. Rejman).

Soil distribution in transect that crossed the study area is an evidence of the changes of relief (Figure 8). Soils on slopes and top of the hills were eroded, and depression was filled with accumulated soil material. Maximum reduction of the soil was about 1.25 m, and maximum accumulation 1.45 m. At the distance of 85 m, the difference of relative height decreased from 4 m of the past relief to 1.5 m at present. Within stand 1, slightly eroded soil prevailed and changed to noneroded soil at the distance of 14 m. Depositional soils that overlaid the buried soil of full sequence of horizons typical for Haplic Luvisol were present in stand 2, and the depth of accumulation was 1.5 m. Within stand 3, noneroded soils were present at the transect distance of 45–48 m and changed to moderately eroded at the end of the stand. Large differentiation of soil depth within a relatively small distance was observed within stand 4 (Figure 9). Soil distribution pointed that the area of this stand was a part of depression of the past relief. The depression vanished during the agricultural use of the area and erosion, and the changes in soil depth are the only evidence of its existence. An inversion of topography was the result of transformation of relief in the stand. Past slopes of SSW aspect were inverted to present NNE slopes, and vice versa. The center of depression is located at present border of watershed. The changes in soil cover within stand 4 were not reflected in the reconstructed map, as sampling points were located in positions that omitted the centre of depression. Localization of transect within the study area was presented in Figure 1.

## 4. Discussion

Studies showed that soils were largely modified after 185 years of arable land use of the studied area. Noneroded soils were scarce (14% of profiles), depositional soil more frequent (30%), and soils of eroded profiles the most frequent (56%). Such large differentiation in soils seems to be typical for some part of loess area of the Lublin Upland. The percentage of noneroded soils ranged from 10 to 21%, eroded soils from 41 to 48%, and depositional soils from 31 to 49% in two other sites of the Upland [5, 7]. Different distribution of soils was reported by Rodzik et al. [34] with larger percentage of noneroded soils that amounted to 35% and smaller percentage of depositional soils that amounted to 18%. The difference could be related to topography of the studied areas. The former studies were performed at the area that consisted of hill's top, slope of southern exposition, and part of valley, and the latter were limited to the slope of northern exposition.

Modification of soils due to erosion and accumulation affected the content of textural fractions and SOC concentration in both the ploughed layer and subsoil. The most significant changes concerned the content of clay. In the ploughed layer, the mean clay concentration in noneroded soil was 88 g kg^−1^ and increased with reduction of profile to the value of 165 in severely eroded soil. Within a subsoil, clay content in noneroded soil was 100 g kg^−1^ and increased to 212 in slightly eroded soils and then decreased to the value of 130 g kg^−1^ in severely eroded soil. The changes well corresponded to the vertical distribution of clay in the profile of noneroded Haplic Luvisol. Similar changes of clay content in noneroded and eroded soils were reported by Turski et al. [5]. The changes are related with excavation of the illuvial horizon B due to erosion and its gradual contribution to the plowed layer. Larger changes are reported in soils of the postglacial area. Heckrath et al. [3] found that clay content in the ploughed layer ranged from 10 to 60%. Similar large differences were observed in the areas of shallow loess that covered clay-reach bedrock [12, 35].

Generally, the reaccumulation of soil material due to water erosion concerns at first the fine material, so higher content of SOC and clay should be observed in the depositional soils. Concentration of SOC follows this tendency, whereas clay content, although higher in the plowed layer of depositional soils, is similar in subsoil layer of noneroded and depositional soils. The explanation could be that some part of the soil material (12%) was removed from the studied area and some part of clay could be washed down the profile of depositional soils. The latter could take place especially in depressions, where stagnation of water was usually observed during the snow melting. The vertical movement of clay particles could follow the same way as pedogenic processes that provided the enrichment of clay in the illuvial Bt horizon. However this hypothesis needs to be confirmed in further studies.

Distribution of soil solum, soil texture fractions, and SOC showed a spatial structure of variability best described by isotropic spherical models of semivariance with a range of autocorrelation of 30–40 m, about half of the distance between the hills of the past relief. Miller et al. [11] showed a similar close relation of range of spatial dependence of soil properties and average distance between hills.

High percentage of soils with eroded profiles is an evidence of strong erosion. However, the calculated annual soil erosion rates were not large and amounted to 1.41 mm ha^−1^. The calculated rate is smaller than the range of annual erosion (2–7 mm) in the Lublin Upland [36–39]. The pattern of soil redistribution with the zones of eroded soils is separated by depositional soils fits to the areas where soil translocation due to tillage is a dominant part of erosion [40, 41]. The measurements of water erosion on runoff plots of various lengths also point to supremacy of soil transport on short distance in the studied area with annual rates up to 5 mm [42, 43]. It seems that both processes of tillage and water erosion participated in reduction of pedon. Measurements of deposited soil material showed that a majority of the eroded soil was maintained within the study area. Similar amounts of erosion and deposition resulted in large smoothing of the studied area, and the average slope decreased by about a half, that is, from 4.3 to 2.2° after 185 years of arable land use. The final effect of erosion and accumulation processes would be transformation of the complex sloping area into a uniform gentle slope. Maruszczak and Uziak [44] found such stadium of transformation of soil catena in loess area of the Lublin Upland.

## 5. Conclusions

The results of the study showed that the change of land use from forest to arable area introduced significant changes to relief and soils in loess area. After about 185 years of cultivation, the relief was largely smoothed, and majority of soil pedons were reduced due to erosion or overbuilt by accumulation. Soils of noneroded pedons were scarce and maintained mainly at the lower part of the slopes at the places of equilibrium between erosion and deposition. Total soil erosion was assessed on 4394 Mg, and 88% from that amount was deposited inside the studied area. Such large deposition resulted in transformation of slopes. The average slope inclination decreased from 4.3 to 2.3°, and slopes above 5° (that constituted 30% of the past topography) vanished at present.

The modification of pedons resulted in large variation of soil properties in both the plowed layer and subsoil, and development of spatial structure of distribution of soil textural fractions and soil organic carbon. Spherical isotropic models of semivariance best described the spatial pattern of soil attributes, and ranges of autocorrelation were 30–40 m, about half the distance between the hills of the past relief. The study proved that present-day relatively flat loess areas had much larger differentiated relief in the past, and distribution of soil solum is the only evidence of the changes.

## Figures and Tables

**Table 1:** Mean depth and standard deviation (SD) of soil horizons of noneroded and eroded soils.

Soil	Soil horizon											
	Ap		E		Bt1		Bt2		BC		C	
	Mean	SD	Mean	SD	Mean	SD	Mean	SD	Mean	SD	Mean	SD
	(m)											
Noneroded (*n* = 25)	0.27	0.02	0.14	0.07	0.42	0.11	0.34	0.11	0.37	0.07	0.11	0.03
Slightly eroded (*n* = 38)	0.27	0.02	—	—	0.22	0.11	0.32	0.11	0.34	0.10	0.10	0.04
Moderately eroded (*n* = 15)	0.25	0.02	—	—	—	—	0.20	0.10	0.29	0.08	0.08	0.02
Severely eroded (*n* = 11)	0.27	0.03	—	—	—	—	—	—	0.27	0.10	0.10	0.05

**Table 2:** Number and mean depth (m) of horizons of depositional soils (standard deviation in brackets).

Depositional soils	Sequence of horizons	*n*	Soil horizon							Solum
			Ap	C1	Ab	E	Bt1	Bt2	BC	Ap-BC
			(m)							
Initial	Ap-Ab-E-Bt1-Bt2-BC-C-Ck	15	0.27	—	0.10	0.14	0.52	0.42	0.37	1.82
			(0.02)	—	(0.05)	(0.09)	(0.15)	(0.12)	(0.06)	(0.22)

Typical	Ap-C1-Ab-E-Bt1-Bt2-BC-C-Ck	28	0.26	0.53	0.14	0.16	0.50	0.65	0.37	2.58
			(0.02)	(0.42)	(0.07)	(0.06)	(0.14)	(0.29)	(0.05)	(0.68)

Disturbed	Ap-C1-E-Bt1-Bt2-BC-C-Ck	1	0.28	0.56	—	0.14	0.24	0.42	0.42	2.06
			(0.00)	(0.00)	—	(0.00)	(0.00)	(0.00)	(0.00)	(0.00)

	Ap-C1-Bt1-Bt2-BC-C-Ck	2	0.26	0.22	—	—	0.35	0.50	0.37	1.69
			(0.00)	(0.13)	—	—	(0.24)	(0.19)	(0.01)	(0.07)

**Table 3:** Soil particle fractions and SOC concentration in the plowed layer and subsoil of the studied soils and results of ANOVA; means were separated by Tukey's HSD test.

Soil	Sand (2–0.05 mm)		Silt (0.05–0.002 mm)		Clay (<0.002 mm)		SOC	
	Mean	CV	Mean	CV	Mean	CV	Mean	CV
	g/kg	%	g/kg	%	g/kg	%	g/kg	%
	Plowed layer							
Noneroded (*n* = 5)	148	8	764^a^	1	88^a^	13	9.6^ab^	7.3
Slightly eroded (*n* = 13)	158	4	701^b^	3	142^b^	13	8.8^b^	12.5
Moderately eroded (*n* = 4)	165	9	688^b^	2	148^b^	14	9.5^ab^	13.9
Severely eroded (*n* = 2)	150	0	685^b^	1	165^b^	3	7.9^b^	9.1
Depositional (*n* = 15)	160	7	743^a^	24	97^a^	24	10.9^a^	11.9

	Subsoil							
Noneroded (*n* = 5)	154	3	752^a^	3	100^a^	28	3.6^a^	7.6
Slightly eroded (*n* = 13)	161	8	629^bc^	4	212^bc^	9	3.4^a^	22.3
Moderately eroded (*n* = 4)	163	3	650^bc^	4	188^bc^	13	3.3^a^	25.5
Severely eroded (*n* = 2)	160	13	710^ab^	3	130^ab^	0	3.0^a^	4.0
Depositional (*n* = 15)	159	6	741^a^	6	100^a^	44	7.6^b^	36.6

Mean values of solum followed by different letters are significantly different at *P* ≤ 0.05.

**Table 4:** Statistics of soil properties and parameters of isotropic spherical models of semivariance.

Soil property	Soil layer	Min.	Max.	Mean	CV	Skewness	Kurtosis	Nugget	Sill	Range of autocorrelation	Anisotropy ratio
			(g kg^−1^)		(%)			(g kg^−1^)^2^		(m)	
Sand	Plowed	140	180	157.7	6.4	0.32	−0.43	0.1	103	36.0	1.50
	Subsoil	140	180	159.5	6.9	0.34	−0.32	52.9	278	310.6	2.22

Silt	Plowed	670	780	721.5	4.6	0.12	−1.26	1	1121	37.6	1.42
	Subsoil	590	780	690.8	9.0	0.04	−1.55	10	3912	32.2	1.41

Clay	Plowed	70	180	120.8	27.4	0.04	−1.19	1	1119	31.6	1.47
	Subsoil	70	240	147.2	41.9	0.03	−1.66	10	3773	26.8	1.31

SOC	Plowed	6.7	13.7	9.7	15.6	0.28	−0.13	0.23	2.39	41.5	1.49
	Subsoil	1.2	10.8	5.0	55.5	1.05	−0.42	0.0001	0.27	31.6	1.41

**Table 5:** Depth of soil horizons in noneroded (apart from Ap horizon) and buried soils, depth of soil solum, and results of ANOVA; means were separated by Tukey's HSD test.

Soil position and number of profiles	Depth of horizons										Depth of solum	
	Ab		E		Bt1		Bt2		BC		Ab-BC	
	Mean	CV	Mean	CV	Mean	CV	Mean	CV	Mean	CV	Mean	CV
	(m)	(%)	(m)	(%)	(m)	(%)	(m)	(%)	(m)	(%)	(m)	(%)
Top (*n* = 2)	0.11	22.2	0.09	7.7	0.26	13.7	0.29	34.5	0.30	20.0	1.04^a^	8.7
Flat area (*n* = 10)	0.10	43.6	0.19	33.7	0.56	27.9	0.43	17.2	0.36	21.1	1.64^b^	6.2
Slope-aspect S (*n* = 14)	0.09	27.5	0.11	43.2	0.42	28.8	0.34	25.0	0.35	16.5	1.31^a^	10.3
Slope-aspect N (*n* = 18)	0.12	16.7	0.14	59.9	0.48	18.8	0.41	29.2	0.38	16.3	1.52^b^	8.8
Floor (*n* = 16)	0.16	43.8	0.18	33.0	0.51	26.8	0.80	34.0	0.40	6.2	2.05^c^	15.7

Mean values of solum followed by different letters are significantly different at *P* ≤ 0.05.
